# Supplementary material for: An assessment of the species diversity and disease potential of Pythium communities in Europe
Source: Nat Commun. 2024 Sep 27;15:8369. doi: 10.1038/s41467-024-52761-0 (PMC11437173; doi:10.1038/s41467-024-52761-0)
Supplement: Supplementary file 1 — Supplementary Information [file 41467_2024_52761_MOESM1_ESM.pdf]

Table S1: Preparation of BARP-Medium. Protocol for preparation of 750 ml BARP medium, developed by Syngenta Agro GmbH (Frankfurt am Main. Germany).

Step	Proceeding
a)	15 g agar (Carl Roth, Karlsruhe, Germany) + 720 ml deionized water + 30 ml tomato vegetable juice with sea salt (Edeka Bio, Hamburg, Germany) + 37.5 mg benomyl (Sigma-Aldrich, St. Louis, USA) + 75 mg pentachloronitrobenzol (Sigma-Aldrich, St. Louis, USA
b)	autoclaving of the mixture
c)	dissolve 1.5 g ampicillin (Carl Roth, Karlsruhe, Germany) in 20 ml deionized water and 1 g rifampicin (Carl Roth, Karlsruhe, Germany) in 67 ml methanol
d)	cool the medium to 40 °C and add 1.5 ml each of the ampicillin and rifampicin solutions
e)	filling the 15 ml tubes with the medium

Table S2: Geographic location of all sampled sites. Metadata of the sampled sites (2019, 2020, 2021). Shown are the Id, country, year, soil texture class, longitude and latitude of the respective sample.

Id	Country	Year	Soil texture class	Longitude	Latitude
P19_L01	France	2019	light	47.6	-0.5
P19_L02	France	2019	heavy	43.8	1.3
P19_L03	France	2019	medium	48.6	7.7
P19_L04	France	2019	medium	50.3	3.0
P19_L05	France	2019	light	49.2	1.2
P19_L06	Italy	2019	light	45.3	9.7
P19_L07	Italy	2019	medium	45.5	8.5
P19_L08	Italy	2019	medium	44.7	7.5
P19_L09	Italy	2019	medium	45.2	7.9
P19_L10	Italy	2019	medium	45.7	11.6
P19_L11	Germany	2019	medium	52.1	8.7
P19_L12	Germany	2019	medium	48.9	12.2
P19_L13	Germany	2019	light	54.3	10.0
P19_L14	Germany	2019	light	54.2	9.6
P19_L15	Germany	2019	medium	51.2	13.3
P19_L16	Germany	2019	light	53.2	11.7
P19_L17	Germany	2019	medium	48.8	12.8
P19_L18	Germany	2019	medium	49.7	8.5
P19_L19	Germany	2019	medium	48.7	8.1
P19_L20	Germany	2019	light	52.5	8.7
P19_L21	Netherlands	2019	light	51.6	4.7
P19_L22	Netherlands	2019	light	51.6	4.7
P19_L23	Belgium	2019	light	50.9	3.1
P19_L24	Belgium	2019	medium	51.1	3.3
P19_L25	Belgium	2019	medium	51.0	3.3
P19_L26	Belgium	2019	medium	51.0	3.4
P19_L27	Spain	2019	medium	42.1	2.4
P19_L28	Spain	2019	medium	41.8	1.0
P19_L29	Spain	2019	medium	42.6	-7.5
P19_L30	Spain	2019	medium	40.9	-5.4
P19_L31	Spain	2019	medium	43.5	-7.3
P19_L32	Romania	2019	heavy	44.1	24.4
P19_L33	Romania	2019	heavy	44.0	28.4
P19_L34	Romania	2019	heavy	47.0	26.9
P19_L35	Romania	2019	heavy	45.6	28.0
P19_L36	Romania	2019	heavy	45.8	21.0
P19_L37	Hungary	2019	heavy	47.4	21.3
P19_L38	Hungary	2019	heavy	46.9	18.7
P19_L39	Hungary	2019	medium	45.8	18.6
P19_L40	Hungary	2019	medium	47.8	17.4
P19_L41	Hungary	2019	medium	47.3	19.3
P19_L42	Austria	2019	medium	48.2	15.6
P19_L43	Austria	2019	medium	47.1	16.0

P19_L44	Switzerland	2019	light	47.6	7.8
P19_L45	Switzerland	2019	medium	46.3	6.9
P20_L01	France	2020	light	47.6	-0.5
P20_L02	France	2020	medium	43.9	1.4
P20_L03	France	2020	medium	50.2	3.0
P20_L04	France	2020	heavy	48.4	7.7
P20_L05	France	2020	light	49.3	1.1
P20_L06	Italy	2020	medium	44.7	7.4
P20_L07	Italy	2020	medium	45.5	8.5
P20_L08	Italy	2020	medium	45.3	7.9
P20_L09	Italy	2020	medium	45.7	11.6
P20_L10	Italy	2020	heavy	45.7	11.6
P20_L13	Germany	2020	light	54.3	10.0
P20_L14	Germany	2020	light	54.2	9.6
P20_L15	Germany	2020	medium	51.2	13.2
P20_L16	Germany	2020	light	53.2	11.7
P20_L17	Germany	2020	medium	48.8	12.8
P20_L19	Germany	2020	medium	48.7	8.1
P20_L20	Germany	2020	light	52.3	8.4
P20_L24	Belgium	2020	light	51.1	3.2
P20_L25	Belgium	2020	light	51.1	3.4
P20_L26	Belgium	2020	light	51.0	3.0
P20_L27	Spain	2020	medium	42.1	2.4
P20_L28	Spain	2020	medium	41.8	1.0
P20_L29	Spain	2020	light	42.6	-7.5
P20_L30	Spain	2020	medium	40.9	-5.5
P20_L31	Spain	2020	medium	43.5	-7.3
P20_L32	Romania	2020	heavy	44.7	23.2
P20_L33	Romania	2020	heavy	44.2	27.2
P20_L34	Romania	2020	heavy	47.1	27.2
P20_L36	Romania	2020	heavy	45.8	21.6
P20_L37	Hungary	2020	heavy	47.4	21.3
P20_L38	Hungary	2020	heavy	47.9	17.4
P20_L39	Hungary	2020	heavy	46.4	18.5
P20_L40	Hungary	2020	heavy	47.1	17.5
P20_L41	Hungary	2020	heavy	47.3	19.2
P20_L42	Austria	2020	light	48.2	14.6
P20_L43	Austria	2020	medium	47.1	16.0
P20_L44	Switzerland	2020	light	47.3	7.5
P20_L45	Switzerland	2020	medium	46.3	6.9
P21_L01	France	2021	light	47.6	-0.5
P21_L06	Italy	2021	heavy	45.3	7.9
P21_L07	Italy	2021	heavy	45.3	7.9
P21_L08	Italy	2021	heavy	44.7	7.4
P21_L09	Italy	2021	heavy	44.4	7.3
P21_L10	Italy	2021	medium	45.7	11.6
P21_L11	Belgium	2021	medium	51.0	3.4

P21_L12	Belgium	2021	medium	51.0	3.3
P21_L13	Germany	2021	heavy	54.3	10.0
P21_L14	Germany	2021	light	54.2	9.6
P21_L15	Germany	2021	medium	54.2	13.3
P21_L16	Germany	2021	light	53.2	11.7
P21_L17	Germany	2021	medium	48.8	12.8
P21_L18	Germany	2021	light	49.4	8.3
P21_L19	Germany	2021	medium	48.7	8.1
P21_L20	Germany	2021	medium	52.3	8.4
P21_L21	Netherlands	2021	medium	52.7	5.0
P21_L22	Belgium	2021	medium	50.9	3.0
P21_L23	Netherlands	2021	medium	52.8	5.0
P21_L24	Belgium	2021	medium	51.1	3.3
P21_L25	Belgium	2021	medium	51.1	3.3
P21_L26	France	2021	medium	44.9	5.0
P21_L27	Spain	2021	medium	42.1	-2.4
P21_L28	Spain	2021	medium	41.8	1.0
P21_L29	Spain	2021	medium	42.6	-7.5
P21_L30	Spain	2021	heavy	43.5	-7.3
P21_L31	Spain	2021	heavy	42.9	-7.9
P21_L32	Romania	2021	medium	44.7	24.3
P21_L33	Romania	2021	heavy	44.3	27.3
P21_L34	Romania	2021	medium	47.4	27.0
P21_L35	Romania	2021	heavy	44.5	28.2
P21_L36	Romania	2021	heavy	45.8	21.7
P21_L37	Hungary	2021	heavy	47.3	19.3
P21_L38	Hungary	2021	heavy	47.4	21.3
P21_L39	Hungary	2021	heavy	47.6	17.2
P21_L40	Hungary	2021	heavy	47.4	17.2
P21_L41	Hungary	2021	heavy	47.0	18.4
P21_L42	Austria	2021	medium	48.2	15.6
P21_L43	Austria	2021	heavy	47.1	16.0
P21_L44	Switzerland	2021	heavy	47.6	8.0
P21_L45	Switzerland	2021	light	47.3	7.5
P21_L46	Czech_rep.	2021	light	49.3	17.4
P21_L48	Germany	2021	heavy	49.4	8.3
P21_L49	Germany	2021	heavy	49.4	8.4

Table S3: Procedure of isolate genomic DNA of bait and soil samples. Genomic DNA was isolated from each 8 x 10 ml of bait media or a pooled 5 g soil sample using CTAB according to a modified method[1].

Step	Bait (8 x 10 ml)	Soil (pooled 5 g)
a)	The samples were stored overnight at -20 °C	Preheat water bath and CTAB solution to 65 °C
b)	Each sample is slightly defrosted and filled into a 50 ml tube, which is filled up to 50 ml with NTI binding buffer	Weigh 5 g soil sample under the sterile bench into a 50 ml Sarstedt tube (5 g soil is a composite sample from the collected subsamples A-H)
c)	Storing for 1 hour at 65 °C	Add 20 ml 2xCTAB solution and 260 µl ß-mercaptoethanol.
d)	Transfer 20 ml into a new 50 ml tube	Vortex briefly
e)	Store sample overnight at -20 °C, then defrost again	Incubate for 90 min at 65 °C in a water bath
f)	Addition of: 20 ml CTAB, 2 ml CTAB (10 x concentration), 500 µl DTT, 200 µl Protenase-K	Cool down Sarstedt tubes on ice for approx. 5 min until they are about hand-warm
g)	Mix sample and store at 65 °C for 1 hour	Option: Add 10 µl RNase A (1 ng/ul) and incubate at 37 °C for 30 min
h)	Centrifuge for 2 minutes at 3.800 rpm	Add 20 ml chloroform-isoamyl alcohol 24:1 and shake upside down for 10 min
i)	Transfer 15 ml into a new 50 ml tube	Centrifuge at 10.000 x g for 20 min.
j)	Addition of 15 ml PCI; mix well	Transfer the upper clear phase into a new Sarstedt tube
k)	Place on overhead shaker for 10 minutes	Add equal amount of ice-cold isopropanol and invert carefully
l)	Centrifuge for 10 minutes at 3.800 rpm	DNA precipitation at one hour on ice
m)	Transfer 7 ml into a new 50 ml tube	Centrifuge for 5 min at 10.000g
n)	Addition of 8 ml NTI-binding-buffer to the 50 ml Tube	Pour off supernatant
o)	Transfer to MiDi M+N column	Wash pellet in 5 ml washing solution I for 20 min (swirl a little and leave in place at room temperature)
p)	Centrifuge for 2 minutes at 3.800 rpm	Centrifuge for 5 min at 10.000g
q)	Addition of 6 ml wash buffer (NT3/EtOH)	Pour off overlap
r)	Centrifuge for 2 minutes at 3.800 rpm	Wash pellet in 5 ml washing solution II for 20 min (swirl a little and leave in place at room temperature)
s)	Addition of 6 ml wash buffer (NT3/EtOH)	Centrifuge for 5 min at 10.000g
t)	Centrifuge for 2 minutes at 3.800 rpm	Dry pellet at room temperature (approx. 10-20 min, depending on pellet size)
u)	Addition of 200 µl Low-TE	Add 250-300 µl TE buffer (depending on pellet size) and resuspend overnight at 4 °C on shaker at 170 rpm
v)	Transfer the sample into a 0.5 ml tube	Storage of DNA at 4 °C
w)	Storage of DNA at 4 °C	

Table S4: Procedure to isolate genomic DNA of *Pythium* sl. isolates. Protocol for gDNA Isolation of *Pythium* s.l. mycelia with Cetyltrimethyl-ammoniumbromide (CTAB) according to a modified method of[1].

Step	Proceeding
a)	Preheat water bath to 65 °C and heat the CTAB / β-mercaptoethanol master mix; Prepare Chloroform / isoamyl mix (24:1)
b)	Add some silica beads and mycelia in 2 ml tube and homogenize for 45 s at 3.500 rpm
c)	Add 1.15 ml of 2xCTAB + 3 µl of β-mercaptoethanol
d)	Incubation in water bath, 1 h, 65 °C
e)	Allow to cool briefly
f)	Add 2 µl RNAse A (1 ng/µl) and incubate at 37 °C for 30 min
g)	Add 500 µl of chloroform-isoamyl alcohol (24:1)
h)	10 min overhead shaker
i)	Centrifugation (10 min, RT, 14.000 rpm)
j)	Transfer the supernatant to a new clean 2 ml tube
k)	Add 700 µl of isopropanol (precooled)
l)	Invert several times
m)	Incubation on ice for 30 min
n)	Centrifugation (10 min, room temperature, 14.000 rpm)
o)	Discard supernatant
p)	Add 1 ml of wash solution I for 10 min
q)	Centrifugation (4 min, room temperature, 14.000 rpm)
r)	Discard the supernatant
s)	Add 500 µl of wash solution II for 10 min
t)	Centrifugation (4 min, room temperature, 14.000 rpm)
u)	Discard the supernatant
v)	Dry pellet at room temperature
w)	Resolve with ddH2O (20-50 µl) on shaker overnight

Table S5: Nucleotide sequences of all primers applied in gene expression profiling. Primers used for quantitative reverse transcription polymerase chain reaction (qRT-PCR) analysis, species identification (Sanger-sequencing) and next-generation sequencing (NGS).

**Name**	**Sequence (5'-3')**
ZmPR1-f	AACAATGGCACCGAGGCT
ZmPR1-r	GTAGTCCTGCGGCGAGTT
ZmPDF1.2-f	CCTCGTCCTCATGCTCCTCC
ZmPDF1.2-r	AAGTTCTCGGTCTGGCACACGTT
ZmETR2-f	GAAGCAAGCATGAAGCAGGG
ZmETR2-r	CAACTCTCTTCGACCCCACC
ZmNCED3-f	GTTGTTCACTCATGCCAAGCA
ZmNCED3-r	GAGTGTGTACGAGAACCAAATGAAA
ZmActin-f	CGACTGCTGAGCGAGAA
ZmActin-r	TGAAGGATGGCTGGAATA
pyth_f	TGCGGAAGGATCATTACCACAC
pyth_r	GCGTTCAAAATTTCGATGACTC

Table S6: Year to year comparison of the most abundant species. Statistical comparison of the three sampling years 2019, 2020 and 2021 of the 10 most abundant species, *G. apiculatum*, *G. attrantheridium*, *G. heterothallicum*, *G. intermedium*, *G. rostratifingens*, *G. sylvaticum* and *G. ultimum.* var. *ultimum, P.* aff. *hydnosporum, P. arrhenomanes* and *P. monospermum*. Statistical differences were tested two sided by a multiple contrast test for *** p < 0.001, ** p < 0.01, * p < 0.05 (degrees of freedom = 112), N = 127.

Species	Comparing	Estimate	std. Error	t-value	p-value
G. apiculatum	2019 - 2020 == 0	-0.00582	0.01161	-0.50183	0.87049
G. apiculatum	2019 - 2021 == 0	-0.00997	0.01104	-0.90267	0.63957
G. apiculatum	2020 - 2021 == 0	-0.00414	0.01181	-0.35084	0.93439
G. attrantheridium	2019 - 2020 == 0	-0.03051	0.04364	-0.69907	0.76437
G. attrantheridium	2019 - 2021 == 0	-0.03978	0.04153	-0.95791	0.60471
G. attrantheridium	2020 - 2021 == 0	-0.00648	0.04297	-0.15082	0.98753
G. heterothallicum	2019 - 2020 == 0	0.06541	0.05523	1.18433	0.46474
G. heterothallicum	2019 - 2021 == 0	0.00889	0.05256	0.16915	0.98434
G. heterothallicum	2020 - 2021 == 0	-0.05652	0.05622	-1.00527	0.57489
G. intermedium	2019 - 2020 == 0	-0.00772	0.02104	-0.36689	0.92849
G. intermedium	2019 - 2021 == 0	-0.02283	0.02002	-1.14076	0.49096
G. intermedium	2020 - 2021 == 0	-0.01512	0.02141	-0.70595	0.76031
G. rostratifingens	2019 - 2020 == 0	-0.03259	0.02311	-1.40985	0.33905
G. rostratifingens	2019 - 2021 == 0	-0.02009	0.02199	-0.91345	0.63277
G. rostratifingens	2020 - 2021 == 0	0.01249	0.02353	0.53104	0.85617
G. sylvaticum	2019 - 2020 == 0	-0.12144	0.03362	-3.61225	0.00127**
G. sylvaticum	2019 - 2021 == 0	-0.00806	0.03199	-0.25179	0.96564
G. sylvaticum	2020 - 2021 == 0	0.11339	0.03422	3.313	0.00352**
G. ultimum. var. *ultimum*	2019 - 2020 == 0	-0.00404	0.03203	-0.12611	0.99126
G. ultimum. var. *ultimum*	2019 - 2021 == 0	0.0313	0.03048	1.02676	0.56137
G. ultimum. var. *ultimum*	2020 - 2021 == 0	0.03534	0.03261	1.08368	0.52591
P. aff. *hydnosporum*	2019 - 2020 == 0	-0.01612	0.03364	-0.47914	0.88121
P. aff. *hydnosporum*	2019 - 2021 == 0	-0.03906	0.03201	-1.22038	0.44348
P. aff. *hydnosporum*	2020 - 2021 == 0	-0.02295	0.03424	-0.67012	0.78116
P. arrhenomanes	2019 - 2020 == 0	0.04635	0.03992	1.16123	0.47855
P. arrhenomanes	2019 - 2021 == 0	0.02716	0.03799	0.71493	0.75503
P. arrhenomanes	2020 - 2021 == 0	-0.0192	0.04064	-0.47239	0.88433
P. monospermum	2019 - 2020 == 0	0.0704	0.01892	3.7215	0.00089***
P. monospermum	2019 - 2021 == 0	0.06015	0.018	3.34147	0.00318**
P. monospermum	2020 - 2021 == 0	-0.01025	0.01926	-0.53215	0.85562

Table S7: Country wise comparison of the most abundant species. Statistical comparison of the 10 sampled countries Austria, Belgium, France, Germany, Hungary, Italy, Netherlands, Romania, Spain, Switzerland, with the grand Mean of all countries pooled for the 10 most abundant species, *G. apiculatum*, *G. attrantheridium*, *G. heterothallicum*, *G. intermedium*, *G. rostratifingens*, *G. sylvaticum* and *G. ultimum.* var. *ultimum*, *P.* aff. *hydnosporum*, *P. arrhenomanes* and *P. monospermum*. Statistically differences compared to the grand Mean were tested two-sided by a multiple contrast for *** p < 0.001, ** p < 0.01, * p < 0.05, ° p < 0.1 (degrees of freedom = 112), N = 126.

Species	Comparing	Estimate	Std. Error	t-value	p-value
G. apiculatum	Austria == 0	-0.00797	0.02021	-0.39425	0.99999
G. apiculatum	Belgium == 0	0.00606	0.01505	0.40239	0.99999
G. apiculatum	France == 0	-0.00776	0.01526	-0.50838	0.99991
G. apiculatum	Germany == 0	0.031	0.01123	276.079	0.06896
G. apiculatum	Hungary == 0	-0.0093	0.01418	-0.65608	0.99916
G. apiculatum	Italy == 0	-0.01055	0.01418	-0.74437	0.99758
G. apiculatum	Netherland == 0	0.05	0.02441	204.826	0.35977
G. apiculatum	Romania == 0	-0.01166	0.01418	-0.82269	0.99461
G. apiculatum	Spain == 0	-0.0117	0.01383	-0.84633	0.99329
G. apiculatum	Switzerland == 0	-0.0117	0.02021	-0.57896	0.99972
G. attrantheridium	Austria == 0	0.15784	0.07601	207.667	0.34121
G. attrantheridium	Belgium == 0	-0.10343	0.0566	-182.745	0.51941
G. attrantheridium	France == 0	0.04391	0.05737	0.76532	0.99696
G. attrantheridium	Germany == 0	-0.08715	0.04222	-206.401	0.34923
G. attrantheridium	Hungary == 0	-0.06962	0.05331	-130.605	0.88123
G. attrantheridium	Italy == 0	0.00156	0.05331	0.02921	1
G. attrantheridium	Netherland == 0	-0.12701	0.09178	-13.838	0.83992
G. attrantheridium	Romania == 0	-0.11408	0.05331	-214.017	0.30172
G. attrantheridium	Spain == 0	-0.03121	0.05199	-0.60025	0.99961
G. attrantheridium	Switzerland == 0	-0.00778	0.07601	-0.1023	1
G. heterothallicum	Austria == 0	0.04463	0.09619	0.46396	0.99996
G. heterothallicum	Belgium == 0	-0.1195	0.07163	-166.835	0.64319
G. heterothallicum	France == 0	0.22805	0.0726	314.095	0.02287*
G. heterothallicum	Germany == 0	-0.00136	0.05344	-0.02551	1
G. heterothallicum	Hungary == 0	0.00782	0.06746	0.11592	1
G. heterothallicum	Italy == 0	-0.08904	0.06746	-131.979	0.87437
G. heterothallicum	Netherland == 0	-0.19474	0.11616	-167.656	0.63678
G. heterothallicum	Romania == 0	0.26495	0.06746	392.739	0.0016**
G. heterothallicum	Spain == 0	-0.03811	0.0658	-0.57915	0.99972
G. heterothallicum	Switzerland == 0	0.0419	0.09619	0.43558	0.99998
G. intermedium	Austria == 0	0.01199	0.03664	0.32731	1
G. intermedium	Belgium == 0	0.07033	0.02728	257.787	0.11192
G. intermedium	France == 0	-0.00825	0.02765	-0.29818	1
G. intermedium	Germany == 0	-0.00371	0.02035	-0.18207	1
G. intermedium	Hungary == 0	-0.0024	0.02569	-0.09332	1
G. intermedium	Italy == 0	-0.01393	0.02569	-0.54231	0.99984
G. intermedium	Netherland == 0	0.00029	0.04424	0.00644	1
G. intermedium	Romania == 0	-0.0197	0.02569	-0.76676	0.99692
G. intermedium	Spain == 0	-0.01651	0.02506	-0.65882	0.99913
G. intermedium	Switzerland == 0	0.01407	0.03664	0.38403	0.99999
G. rostratifingens	Austria == 0	-0.00659	0.04025	-0.16363	1
G. rostratifingens	Belgium == 0	0.02063	0.02998	0.68817	0.99874
G. rostratifingens	France == 0	-0.01109	0.03038	-0.36487	1
G. rostratifingens	Germany == 0	0.03447	0.02236	154.124	0.73769
G. rostratifingens	Hungary == 0	-0.01888	0.02823	-0.66888	0.99901

G. rostratifingens	Italy == 0	-0.00801	0.02823	-0.28362	1
G. rostratifingens	Netherland == 0	-0.01138	0.04861	-0.23406	1
G. rostratifingens	Romania == 0	-0.00775	0.02823	-0.27465	1
G. rostratifingens	Spain == 0	0.0134	0.02754	0.48677	0.99994
G. rostratifingens	Switzerland == 0	0.01274	0.04025	0.31659	1
G. sylvaticum	Austria == 0	0.05882	0.05855	100.458	0.97646
G. sylvaticum	Belgium == 0	0.02008	0.0436	0.46053	0.99996
G. sylvaticum	France == 0	-0.03256	0.0442	-0.73672	0.99777
G. sylvaticum	Germany == 0	-0.01351	0.03253	-0.41527	0.99999
G. sylvaticum	Hungary == 0	-0.00938	0.04107	-0.22839	1
G. sylvaticum	Italy == 0	0.03251	0.04107	0.79169	0.99602
G. sylvaticum	Netherland == 0	0.06691	0.07071	0.94634	0.98456
G. sylvaticum	Romania == 0	-0.04039	0.04107	-0.98351	0.97969
G. sylvaticum	Spain == 0	-0.03529	0.04005	-0.88107	0.99088
G. sylvaticum	Switzerland == 0	-0.01471	0.05855	-0.25116	1
G. ultimum. var. *ultimum*	Austria == 0	-0.07704	0.05579	-138.099	0.84153
G. ultimum. var. *ultimum*	Belgium == 0	0.21856	0.04154	526.133	0,00001***
G. ultimum. var. *ultimum*	France == 0	-0.05801	0.04211	-137.778	0.84336
G. ultimum. var. *ultimum*	Germany == 0	-0.03469	0.03099	-111.932	0.95182
G. ultimum. var. *ultimum*	Hungary == 0	0.05925	0.03912	151.446	0.75663
G. ultimum. var. *ultimum*	Italy == 0	-0.08495	0.03912	-217.123	0.28351
G. ultimum. var. *ultimum*	Netherland == 0	0.18771	0.06737	278.648	0.06408
G. ultimum. var. *ultimum*	Romania == 0	-0.06004	0.03912	-153.466	0.74254
G. ultimum. var. *ultimum*	Spain == 0	-0.06443	0.03816	-168.826	0.62779
G. ultimum. var. *ultimum*	Switzerland == 0	-0.06396	0.05579	-11.465	0.94404
P. aff. *hydnosporum*	Austria == 0	-0.05641	0.05859	-0.96281	0.98254
P. aff. *hydnosporum*	Belgium == 0	-0.03313	0.04363	-0.75931	0.99715
P. aff. *hydnosporum*	France == 0	-0.06323	0.04422	-142.978	0.8125
P. aff. *hydnosporum*	Germany == 0	0.0256	0.03255	0.78654	0.99622
P. aff. *hydnosporum*	Hungary == 0	-0.06035	0.04109	-14.688	0.78746
P. aff. *hydnosporum*	Italy == 0	0.0425	0.04109	103.437	0.97125
P. aff. *hydnosporum*	Netherland == 0	0.10279	0.07075	145.287	0.79783
P. aff. *hydnosporum*	Romania == 0	0.00748	0.04109	0.18214	1
P. aff. *hydnosporum*	Spain == 0	0.03384	0.04008	0.84432	0.99341
P. aff. *hydnosporum*	Switzerland == 0	0.00571	0.05859	0.09747	1
P. arrhenomanes	Austria == 0	-0.0097	0.06952	-0.13955	1
P. arrhenomanes	Belgium == 0	0.03509	0.05177	0.67788	0.99889
P. arrhenomanes	France == 0	-0.00698	0.05248	-0.13298	1
P. arrhenomanes	Germany == 0	0.03483	0.03862	0.90176	0.98916
P. arrhenomanes	Hungary == 0	-0.06399	0.04876	-131.235	0.87806
P. arrhenomanes	Italy == 0	-0.05707	0.04876	-11.704	0.93649
P. arrhenomanes	Netherland == 0	-0.07554	0.08395	-0.89974	0.98933
P. arrhenomanes	Romania == 0	0.09987	0.04876	204.813	0.35984
P. arrhenomanes	Spain == 0	0.01999	0.04756	0.42028	0.99998
P. arrhenomanes	Switzerland == 0	0.05427	0.06952	0.78053	0.99644
P. monospermum	Austria == 0	0.00123	0.03295	0.03748	1
P. monospermum	Belgium == 0	-0.02553	0.02453	-104.044	0.97013
P. monospermum	France == 0	0.0286	0.02487	115.024	0.94295
P. monospermum	Germany == 0	-0.02232	0.0183	-121.958	0.91897
P. monospermum	Hungary == 0	-0.0362	0.02311	-156.675	0.71958
P. monospermum	Italy == 0	0.08106	0.02311	350.787	0.00701**
P. monospermum	Netherland == 0	-0.02195	0.03979	-0.5517	0.99981
P. monospermum	Romania == 0	-0.02507	0.02311	-108.496	0.96054
P. monospermum	Spain == 0	-0.00707	0.02254	-0.31365	1
P. monospermum	Switzerland == 0	0.04548	0.03295	138.035	0.84189

Table S8: Characterisation of soil textures. Classification of soil texture class according to clay content or soil type. S = sand, U = silt, T = clay, L = loam, s = sandy, u = silty, t = clayey, l = loamy (* designation of the soil type according to ÖNORM L 105080).

**Soil texture class**	**Clay content**	**Soiltype***
light	< 15 %	S, uS, lS, sU
medium	15 – 25 %	tS, U, lU, sL
heavy	> 25 %	L, uL, sT, lT, T

Table S9: *Pythium* s.l. abundance as a function of soil texture classes. Abundance of *Pythium* s.l. estimates based on soil texture classes (heavy, medium and light) compared to the mean number of *Pythium* s.l. abundance for all sites sampled. Significant differences were tested against the mean of all samples by multiple contrasts for *** $p < 0.001$, ** $p < 0.01$, * $p < 0.05$ (degrees of freedom = 110), N = 127.

Soil texture class	Estimate	Std. Error	t-value	p-value
heavy == 0	-2027.61914	1554.3821	-1.30445	0.39086
light == 0	416.80357	1455.3238	0.2864	0.95511
medium == 0	1610.81558	1097.09015	1.46826	0.30553

Table S10: Detected species of *Pythium* s.l. in dependency of soil texture classes. Estimates of *Pythium* s.l. based on soil texture classes (heavy, medium and light) compared to the mean number of *Pythium* s.l. for all sites sampled. Significant differences were tested against the mean of all samples by multiple contrasts for *** p < 0.001, ** p < 0.01, * p < 0.05, ° p < 0.1 (degrees of freedom = 110), N = 127.

Soil texture class	Estimate	Std. Error	t-value	p-value
heavy == 0	-0.93877	0.66156	-1.41903	0.32995
light == 0	-0.04952	0.6194	-0.07996	0.99642
medium == 0	0.9883	0.46693	2.11657	0.08903°

Table S11: Evaluation of germination capacity after infection. Comparison of the germination rate of corn inoculated with *G. attrantheridium* and *G. ultimum* var. *ultimum* with a non-inoculated control. Statistical differences were tested two sided by a multiple contrast test for *** $p < 0.001$, ** $p < 0.01$, * $p < 0.05$ (normal approximation), n = 6.

**Soil texture class**	**Estimate**	**Std. Error**	**z-value**	**p-value**
G. attrantheridium - NC == 0	0.5498	1.8669	0.295	0.9433
G. ultimum var. *ultimum* - NC == 0	-4.3343	1.6891	-2.566	0.0201*

Table S12: Evaluation of shoot fresh weight after *Pythium* s.l. infection. Comparison of the shoot fresh weight per plant of corn inoculated with *G. attrantheridium* and *G. ultimum var. ultimum* with a non-inoculated control. Statistical differences were tested two sided by a multiple contrast test for *** $p < 0.001$, ** $p < 0.01$, * $p < 0.05$ (degrees of freedom = 15), n = 6.

Soil texture class	Estimate	Std. Error	t-value	p-value
G. attrantheridium - NC == 0	-0.2211	0.5163	-0.428	0.87666
G. ultimum var. *ultimum* - NC == 0	-2.2544	0.5163	-4.366	0.00106**

Table S13: Evaluation of root dry weight after *Pythium* s.l. infection. Comparison of the root dry weight per plant of corn inoculated with *G. attrantheridium* and *G. ultimum* var. *ultimum* with a non-inoculated control (NC). Statistical differences were tested two sided by a multiple contrast test for *** p < 0.001, ** p < 0.01, * p < 0.05 (degrees of freedom = 15), n = 6.

Soil texture class	Estimate	Std. Error	t-value	p-value
G. attrantheridium - NC == 0	-0.02667	0.02503	-1.065	0.4777
G. ultimum var. *ultimum* - NC == 0	-0.07333	0.02503	-2.929	0.0192*

Table S14: Gene expression profiling after *Pythium* s.l. infection. Comparison of gene expression of PR1, PDF1.2, JA, ETR2 and NCED3 in corn seedlings inoculated with *G. attrantheridium* (P2), *G. ultimum* var. *ultimum* (P38) or non-inoculated (NC). Statistical differences were tested two sided by a multiple contrast test for *** p < 0.001, ** p < 0.01, * p < 0.05 (degrees of freedom = 24), n = 3.

***Pythium* species**	**Gene**	**Estimate**	**Std. Error**	**t-value**	**p-value**
NC – P2	ETR2 == 0	-0.28933	0.20476	-1.413	0.655
NC – P38	ETR2 == 0	-0.45379	0.20476	-2.216	0.227
P2 – P38	ETR2 == 0	-0.16446	0.20476	-0.803	0.948
NC – P2	NCED3 == 0	-0.13626	0.20476	-0.665	0.977
NC – P38	NCED3 == 0	-0.37976	0.20476	-1.855	0.391
P2 – P38	NCED3 == 0	-0.2435	0.20476	-1.189	0.788
NC – P2	PDF1.2 == 0	0.06305	0.20476	0.308	1
NC – P38	PDF1.2 == 0	1.25788	0.20476	6.143	<0.001***
P2 – P38	PDF1.2 == 0	1.19483	0.20476	5.835	<0.001***
NC – P2	PR1 == 0	-2.89379	0.20476	-14.132	<0.001***
NC – P38	PR1 == 0	-4.54529	0.20476	-22.198	<0.001***
P2 – P38	PR1 == 0	-1.6515	0.20476	-8.065	<0.001***

References

1. Rogers, S. O. & Bendich, A. J. Extraction of DNA from milligram amounts of fresh, herbarium and mummified plant tissues. *Plant Mol Biol* **5,** 69–76; 10.1007/bf00020088 (1985).
