# Supplementary material for: An assessment of the species diversity and disease potential of Pythium communities in Europe
Source: Nat Commun. 2024 Sep 27;15:8369. doi: 10.1038/s41467-024-52761-0 (PMC11437173; doi:10.1038/s41467-024-52761-0)
Supplement: Supplementary file 3 — Description of Additional Supplementary Files [file 41467_2024_52761_MOESM3_ESM.pdf]

Description of Additional Supplementary Files:

File Name: Supplementary Data 1

Description: The file with the chemicals and materials used in this study.

File Name: Supplementary Data 2

Description: Sources for the scripts, software and original data used to create the figures in this study.
